# Psychophysiological Responsivity to Script-Driven Imagery: An Exploratory Study of the Effects of Eye Movements on Public Speaking Flashforwards

**DOI:** 10.3389/fpsyt.2015.00115

**Published:** 2015-08-14

**Authors:** Michelle Kearns, Iris M. Engelhard

**Affiliations:** ^1^Division of Clinical and Health Psychology, Utrecht University, Utrecht, Netherlands

**Keywords:** flashforwards, eye movements, experiment, heart rate, anxiety, dual-task

## Abstract

A principle characteristic of public speaking anxiety relates to intrusive mental images of potential future disasters. Previous research has found that the self-reported emotionality of such “flashforwards” can be reduced by a cognitively demanding, dual-task (e.g., making eye movements) performed whilst holding the mental image in-mind. The outcome measure in these earlier studies was participants’ self-reported emotional intensity of the mental image. The current study (*N* = 34) explored whether an objective measure of emotionality would yield similar results in students with public speaking anxiety. A script-driven imagery procedure was used to measure psychophysiological responsivity to an audio script depicting a feared (public speaking) scenario before and after an eye movement intervention. Relative to the control condition (imagery only), those who made eye movements whilst holding a mental image of this scenario in-mind demonstrated a significant decrease in heart rate, which acted as a measure of emotionality. These findings add to a previous body of research demonstrating the beneficial qualities of dual-tasks and their potential for treatment of both past and future-oriented anxieties.

## Introduction

Many of us know that feeling of standing in front of a crowded room, all eyes on you, waiting expectantly for enlightenment on your chosen subject matter. Your hands begin to tremble, your heart starts racing, and beads of perspiration form on your forehead. Not many people enjoy public speaking, but for some even the thought of giving a presentation induces intense fear and a wide range of unwanted physiological responses. Public speaking anxiety is one of the most prevalent anxiety disorders today, with estimates suggesting that 75% of individuals experience some degree of unease in public speaking (1). Considering then that oral presentations embody such an important and unavoidable element of student and working life, as well as being of significant importance in terms of employability and upward mobility (2), investigating how this fear can be overcome represents a worthwhile pursuit.

Public speaking anxiety is a specific type of social and communication-based anxiety which can result in physiological arousal, negative self-focused cognitions, and accompanying behavioral demonstrations such as trembling or shaking (3, 4). Often referred to under a wide variety of different labels from speech fear, social speech fright, or speech anxiety to umbrella labels such as stage fright, audience anxiety, or performance anxiety [e.g., in Ref. (5, 6)], this particular phobia has its own individual qualities that contribute to its status as a stand-alone fear. For instance, individuals with speaking anxiety show stronger psychophysiological responses [greater heart rate (HR) response] when delivering a speech compared to those with generalized social anxiety disorder (6). Previous research employing varying methods to provoke anxiety has also demonstrated that the anxiety levels generated by public speaking tasks is generally higher than those for alternate tasks performed in similarly stressful situations [e.g., performing mental arithmetic in front of an audience; (7)]. Bodie (4) outlines how the combination of an audience presence and expectations particular to public speaking provide a situation-specific classification of public speaking anxiety, with apprehension of the threat of unsatisfactory evaluations from a defined public representing a key component of the phobia.

Although at times, a degree of nervousness has been shown to be beneficial to the performance of an individual [e.g., in Ref. (8)], high levels of anxiety can result in poor speech preparation and decision-making, and negatively affect performance [(9); see also in Ref. (10)]. If left untreated, public speaking anxiety will frequently persist and cause individuals to become avoidant, potentially hindering their success at both an academic level and in the workplace (11). Exploring potential intervention methods is therefore of significant importance, with one such method – eye movements targeting mental imagery – forming the primary pillar of investigation in this paper.

### Mental imagery and eye movements

A principle characteristic of public speaking anxiety and performance anxiety in general relates not only to intrusive thoughts and cognitions causing a dip in the execution of a particular tasks [e.g., in Ref. (12, 13)], but also to vivid and intrusive mental images of potential future disasters. Imagery about performing badly and being negatively evaluated has been shown to be commonplace amongst those with social phobia and varying types of anxiety disorders [e.g., in Ref. (14–16)]. One such example demonstrated in previous research is a woman who visualizes her mouth moving but no words coming out, and being perceived as very strange as a result (16). These unwanted flashforwards are similar in nature to “flashbacks” demonstrated by posttraumatic stress disorder (PTSD) patients; negative mental images loaded with sensory information, and resulting in the elicitation of substantial arousal and distress (17). In fact, neurobiological mechanisms that mediate the capacity to retrieve specific memories also mediate one’s capacity to envision the future (18). It has been shown that in some cases mental imagery may evoke greater emotional responses than related verbal cognitions, and thus represents a prime target for interventions (17, 19); an observation that has not been overlooked.

A substantial body of research has previously demonstrated that self-rated vividness and emotionality of flashbacks can be reduced through the engagement of cognitively demanding dual-tasks [for a review, see Ref. (20)]. Dual-task trials, where, for instance, eye movements are made whilst simultaneously holding an image in-mind, are thought to attenuate the sensory qualities of these images as a result of working memory taxation. Indeed, previous studies have shown that dual-tasks that tax working memory are effective [e.g., in Ref. (21)], and tasks that barely tax working memory (such as passively listening to sounds) are not effective (22). Through the process of reconsolidation into long-term store, the memory should become less vivid and less distressing (23–25). Gunter and Bodner (25) found that the effects of memory taxation via eye movements persist for one week. This dual-task practice forms the central component of Eye Movement Desensitization and Reprocessing (EMDR) treatment, which is now recommended as one of the first-line treatment methods for PTSD by several organizations [e.g., in Ref. (26, 27)]. It has recently been established that this dual-task technique is not just effective on past memories however, but that it can also successfully degrade future-oriented images, including flashforwards related to performance anxiety (28, 29). The present study will attempt to build upon these findings by altering the experimental paradigm used to include an objective measure, thus overcoming some limitations of earlier studies relying on self-report alone.

### Adaptation of experimental paradigm

In the majority of previous studies examining the effects of dual-tasks, efficacy of the treatment has been gaged through use of subjective measures – for example, asking participants to report pre- and post-test levels of vividness and emotionality in respect to targeted mental images [e.g., in Ref. (28, 29)]. Though in theory this practice may be sound, as the primary aim of the treatment is to reduce the subjective emotionality of such images, relying wholly on subjective measures can be problematic in some instances. It is possible that self-reports may be affected by experimental demand, and thus sole reliance on such measures may potentially jeopardize the scientific integrity of study findings. The possible limitations of self-report measures in experiments investigating the effects of dual-tasks have already been recognized and addressed in two prior studies. Engelhard et al. (30) found that, relative to those in a recall only condition, startle responses decreased in participants who completed a dual-task. van den Hout et al. (31), meanwhile, used a behavioral reaction time task to demonstrate attenuated vividness of a neutral image in those who made eye movements whilst holding this image in-mind. The current study will attempt to overcome similar demand characteristics by using different objective measures to assess the effectiveness of a dual-task in reducing emotionality toward a feared scenario.

Given that increased arousal has a well-documented relationship with performance anxiety [e.g., in Ref. (3, 4)], physiological reactivity to the feared scenario will be used as a determinant of emotional valence rather than relying on self-report alone. This is a particularly relevant assessment tool for gaging emotional response to imagery, as research has shown that mental imagery elicits physiological responses akin to the arousal experienced upon anticipation of actual exposure to a feared stimulus [e.g., in Ref. (32)].

An additional alteration to the experimental paradigm in the present study relates to the source of target imagery for the dual-task. Pre-recorded audio scripts depicting the feared scenario (speaking in public) will be utilized instead of participants conjuring up their own mental image. The format for presenting these scripts is based upon the script-driven imagery procedure originally used by Lang et al. (12) to investigate physiological reactivity to fear imagery, and perfected by Pitman and his colleagues (33–36) in a series of studies investigating the physiological underpinnings of PTSD. Use of this procedure will eliminate a lack of control over memory recall during self-report gages and ensure that both self-reports of emotional intensity and vividness, and the physiological responses recorded, are directed at the same mental image both before and after the dual-task intervention.

In the current study, eye movements will be used as the cognitively demanding task performed whilst participants engage in mental imagery. Eye movements are just one of the number of methods that have previously been used to good effect in dual-task trials, with other cognitive tasks that have previously been shown to reduce the vividness and emotionality of mental images including auditory shadowing (25), drawing a complex figure (25), attentional breathing (21), playing Tetris (30), and counting (37–39). It is therefore important to note that although this study discusses the effects of eye movements with mental imagery, this term is used to represent the entire dual-task trial process; any potential intervention effects will stem from the taxing of working memory rather than simply the act of making eye movements.

The effectiveness of the eye movement intervention will be primarily determined from differences in pre- vs. post-test physiological measures [i.e., HR; (6)] and self-reports ratings (emotional intensity and vividness). It is expected that participants who have made eye movements whilst simultaneously holding a negative mental image in-mind will give lower subjective self-report ratings of emotional intensity of the mental image and demonstrate attenuated physiological reactivity toward the imagery depicted in the script, indicating a decline in the emotional intensity experienced.

## Materials and Methods

### Participants

The study sample consisted of students from Utrecht University and Hogeschool Utrecht (higher vocational school) who participated in return for course credit or a financial reward. The study was approved by the institutional review board of the Faculty of Social and Behavioural Sciences at Utrecht University. The sample size was set before data analysis, and all measures that were collected are reported. In total, 442 students were administered a six-item screening scale for public anxiety. This was a Dutch Translation of the Public Speaking sub-scale (PSA) of the Personal Report of Communication Apprehension (PRCA-24) (40), which was found to generally produce reliability estimates in the range of 0.80–0.85 (41). Although not originally designed for clinical use, the PRCA-24 has been has since been recommended as a clinical tool (42) and is the most widely used self-report scale of communication apprehension because of its consistent reliability and validity (43, 44). Researchers have suggested the sole use of the PSA sub-scale of the PRCA-24 if the research is concerned with the cognitive trait of public speaking anxiety (4), as is the case in this study. The questionnaire consists of the following items: (1) I have no fear of giving a speech, (2) Certain parts of my body feel very tense and rigid while giving a speech, (3) I feel relaxed while giving a speech, (4) My thoughts become confused and jumbled when I am giving a speech, (5) I face the prospect of giving a speech with confidence, and (6) While giving a speech, I get so nervous I forget facts I really know. Items are rated on a 5-point scale based on the degree to which participants agree with each statement (1 = *strongly disagree* to 5 = *strongly agree*). Items 1, 3, and 5 were reversely scored; for scoring procedure see McCroskey (40). Sixty-eight students with a score of 20 or higher^1^ were invited to take part in the study, with McCroskey (45) advising that those with a score of 18 or higher experience significant apprehension in public speaking. Twenty-three of these declined the offer, primarily due to the main testing period coinciding with the exam and study period of the university. The exclusion criterion was having prior knowledge of EMDR (*n* = 11). The final sample consisted of 34 participants (9 males) with a mean age of 21.4 years (SD = 2.99) and a mean PRCA-24 Public Speaking Sub-score of 22.9 (SD = 2.67, range: 20–30).

### Script preparation

The audio scripts used in this study consisted of Dutch translations of standardized fear (public speaking) and neutral (looking out a living room window) scripts that have been utilized in several previous experiments [e.g., in Ref. (12, 33, 36)]. Each script of approximately 30 s in length was recorded in a neutral voice for playback in the laboratory, and depicted the scenario in detail. For instance, the public speaking script was as follows: “You have volunteered to give a presentation to a class in which you badly need to improve your grade/Your palms have become sweaty, and you tense up the muscles of your forehead/As you walk to the front of the room, you breathe rapidly and glance around at the faces of the audience.” Prior to the main experiment, a pilot study consisting of nine participants with a fear of public speaking was run to ensure that (1) participants demonstrated higher levels of physiological reactivity to the fear (public speaking) script than to the neutral script and (2) participants still demonstrated heightened responses to the fear script on the second time of listening.

### Procedure

#### Laboratory Procedure

The laboratory session consisted of individual testing sessions for each participant in a quiet, light attenuated room. Upon entering they received verbal and written information about the study and signed a consent form. Next, they were asked whether they were familiar with several treatments, including EMDR, and, if so, to describe them. At this point, anyone with prior knowledge of EMDR was excluded from the study and did not complete the experiment. The participant’s skin was cleaned using an alcohol wipe, and electrodes were prepared and attached. Participants were then seated at a desk with personal computer which was used to present all of the stimuli used during the experiment via E-Prime 2.0 Professional software. Once seated comfortably, a relaxation exercise was played.

#### Script-Driven Imagery Procedure

Upon completion of the relaxation exercise, a 30-s recording of baseline physiological responses was taken. This was followed by the presentation of the neutral (looking out a window) and fear (public speaking) scripts in line with the well-established script-driven imagery procedure developed by Lang et al. (12) and Pitman et al. (33). Each script presentation consisted of (1) a 30-s script presentation, (2) a 30-s imagery period where participants were instructed to continue imagining the experience presented in the script from beginning to end, (3) an assessment period that consisted of emotional intensity and vividness visual analog scale (VAS) ratings, and (4) a 30-s recovery period. In all cases, the neutral script was presented prior to the fear script to eliminate the possibility of a carryover of negative affect.

#### Mental Imagery Experiment

After the first script-driven imagery procedure was complete, participants undertook a mental imagery experiment based on the protocol for unpleasant images designed by van den Hout et al. (46) and modified by Engelhard et al. (28, 29) to future-oriented images. Participants were randomly assigned to either the experimental condition or to the control condition, with 17 participants in each group. The experimental condition involved six identical 24-s phases of imagery with eye movements, separated by 10-s breaks during which participants were instructed to think of something else. This set-up mirrors an established paradigm used in several previous studies [e.g., in Ref. (25, 28, 29, 46, 47)]. For each of the six eye movement phases, participants were told to continuously visualize the public speaking scenario (giving a speech in front of their class) that they listened to on the audio recording and at the same time to follow a 1-cm light gray circle with their eyes as it moved horizontally from one side of the screen to the other at a rate of one movement per second, all the while keeping their head still. The control condition followed the same paradigm, but with participants focusing on a stationary rather than a moving circle.

A second presentation of the neutral and fear scripts followed the mental imagery experiment, with an identical format to the script-driven imagery procedure outlined above. The experiment ended with a 4-min relaxation exercise in order to reduce any possible distress that resulted from participation. Participants were then debriefed, thanked, and given their reward (course credit or a small financial reward).

### Measures

#### Physiological Measures

A BIOPAC MP150 system connected to a separate computer running AcqKnowledge 4.1 software was used to measure HR, skin conductance (SC), and electromyograms (EMGs) of the lateral frontalis and corrugator facial muscles. HR was the main measure of interest [see Ref. (6)], and SC and EMG were measured for exploratory purposes. SC level was obtained through 13/8 mm Ag/AgCl electrodes filled with isotonic paste and placed on the volar side of the wrist. The transducer was connected to a BIOPAC-GSR100C module with a sampling rate of 200 times per second. The EDA signal was transformed into microsiemens (μS) units before being analyzed. Activity of the lateral frontalis and corrugator was recorded on the left side of the face using bipolar placements of 7/4 mm Ag/AgCl surface electrodes in accordance with published guidelines (48). The EMG raw signal was measured in microvolts using a BIOPAC-EMG100A module with a sampling frequency of 2000 Hz. Online transformation was used to acquire the integrated [root mean square (RMS)] EMG for both measures. HR was measured with a BIOPAC-PPG100C Pulse Plethysmogram Amplifier via a transducer attached by Velcro to the participant’s index finger. The PPG signal was transformed online to pulse rate, measured in beats per minute, before being analyzed.

#### Self-Report Measures

Self-report measures of emotional intensity and vividness were obtained using two 100 mm VAS. Vividness ratings were collected for exploratory purpose; we did not expect that they would decrease due to the fact that participants would listen to the fear script in its entirety for a second time after completing the mental imagery experiment. Immediately after each imagery period of the script-driven imagery procedure, participants were asked to indicate their subjective feelings toward each script on a scale that ranged from 0 = not vivid (unpleasant) at all to 100 = extremely vivid (unpleasant).

## Results

### Self-reports

**Figure 1:**
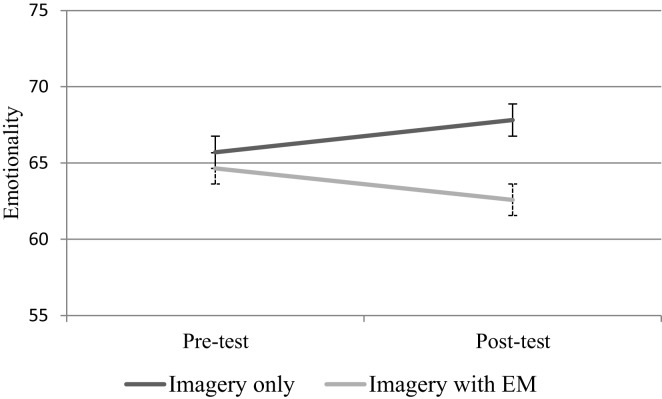
**Mean emotionality ratings with standard error bars for imagery only (*n* = 17) and imagery with eye movements (EM; *n* = 17) conditions**.

**Figure 2:**
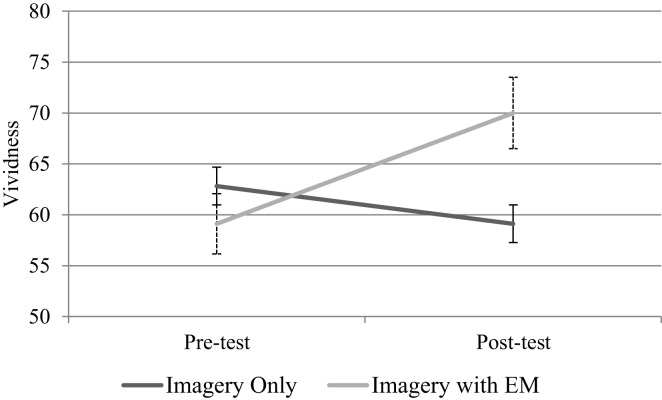
**Mean vividness ratings with standard error bars for imagery only (*n* = 17) and imagery with eye movements (EM; *n* = 17) conditions**.

The mean emotional intensity and vividness ratings are depicted in Figures 1 and 2, respectively. Both of the self-report ratings were analyzed with repeated measures ANOVAs with Condition (imagery with eye movements vs. imagery only) as the between-subjects factor and Time (pre-test vs. post-test) as the within-subjects factor. Emotionality ratings increased from pre-test to post-test level for the imagery only condition, and decreased for the imagery with eye movements condition (see Figure 2), however, neither of the main effects of Time or Condition, or the interaction between Time and Condition proved significant, all *Fs*(1,32) < 1.

Vividness scores were tested for normal distribution and, using the methods outlined by Hoaglin and Iglewicz (49), one outlier was detected and transformed. It was found that neither of the main effects of Time [*F*(1,32) < 1, *p* = 0.50, $\eta_{\text{p}}^{2}=0.02$] or Condition [*F*(1,32) < 1, *p* = 0.42, $\eta_{\text{p}}^{2}=0.02$], or the interaction between Time and Condition [*F*(1,32) = 2.79, *p* = 0.11, $\eta_{\text{p}}^{2}=0.08$] were significant.

### Physiological measures

Pre- and post-test response (change) scores were calculated for the physiological dependent variables by subtracting a baseline mean score from the mean score during imagery (33). Response scores from neutral scripts where a non-stressful scene was visualized served as a control to ensure that the fear script elicited higher levels of physiological arousal, and that this arousal could be attributed to increased anxiety. HR, SC, and EMG of the frontalis and corrugator responses to fear imagery were examined separately using 2 × 2 repeated measures ANOVAs with Condition (Imagery with eye movements vs. imagery only) as the between-subjects factor and Time (pre-test vs. post-test) as the within-subjects factor.

**Figure 3:**
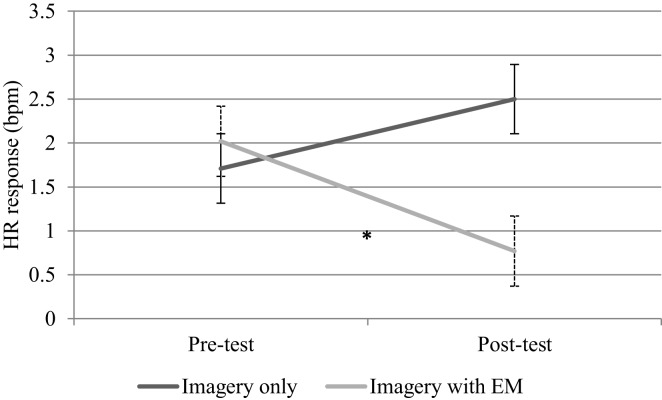
**Mean heart rate (HR) change scores with standard error bars measured in beats per minute (bpm) during imagery of fear (public speaking) script for imagery only (*n* = 17) and imagery with eye movements (EM; *n* = 17) conditions**. *Statistically significant difference between pre- and post-test change scores, *p* < 0.05.

There was no difference in baseline HR scores between the imagery only (*M* = 79.98, SD = 14.53) and imagery with eye movements conditions (*M* = 81.15, SD = 11.60, *t*(32) = 0.70, *p* = 0.49), whilst a paired samples *t*-test revealed that HR response to the fear script was significantly greater than that to the neutral script, *t*(33) = 0.29, *p* = 0.01, in the pre-test reading. Table 1 shows the mean response scores to neutral and fear imagery, and Figure 3 depicts the HR response to fear scripts for pre- and post-test measures. A 2 × 2 ANVOVA with HR response to fear imagery as the dependent variable revealed that neither of the main effects of Time or Condition were significant, both *Fs*(1,32) < 1. As predicted however, the crucial interaction between Time and Condition proved significant, *F*(1,32) = 5.87, *p* = 0.02, $\eta_{\text{p}}^{2}=0.16$. An independent samples *t*-test showed that HR response scores to fear imagery did not differ between conditions in the pre-test, *t*(32) < 1. Paired *t*-tests revealed that response scores decreased significantly from the pre-test to the post-test for the eye movements condition, *t*(16) = 2.73, *p* = 0.01 (one-tailed), but not for the imagery only condition, *t*(16) = 1.18, *p* = 0.13.

**Table 1:** **Mean change scores for physiological measures during imagery of neutral and fear scripts**.

		Imagery only (*n* = 17)		Imagery with EM (*n* = 17)	
		Neutral	Fear	Neutral	Fear
HR	Pre-test	–0.31 (5.39)	1.71 (3.30)	–0.01 (2.90)	2.03 (2.02)
	Post-test	–1.53 (4.10)	2.50 (3.86)	–3.70 (3.45)	0.77 (2.48)
SC	Pre-test	–0.00047 (0.0056)	0.00011 (0.0055)	–0.00902 (0.0380)	–0.00797 (0.0391)
	Post-test	0.00160 (0.0064)	0.00110 (0.0071)	–0.01200 (0.0392)	–0.00810 (0.0390)
EMG frontalis	Pre-test	–0.00100 (0.0020)	–0.00110 (0.0020)	–0.00180 (0.0013)	–0.00183 (0.0013)
	Post-test	–0.00012 (0.0021)	–0.00012 (0.0023)	–0.00191 (0.0019)	–0.00221 (0.0018)
EMG corrugator	Pre-test	0.00021 (0.0012)	0.00021 (0.0014)	–0.00055 (0.0016)	0.00001 (0.0017)
	Post-test	–0.00041 (0.0015)	0.00011 (0.0019)	0.00022 (0.0053)	0.00116 (0.0072)

*SDs of the mean are in parentheses. Heart rate (HR) is measured in beats per minute, skin conductance (SC) is measured in microsiemens and electromyography (EMG) root mean square response scores for the corrugator and frontalis are measured in microvolts*.

Although both SC and EMG of the corrugator demonstrated significantly higher response rates to the fear script than to the neutral script, no other significant results were found for either measure, or for EMG of the frontalis, so they will not be reported; mean response scores are presented in Table 1.

## Discussion

Previous studies have found that taxing working memory with dual-tasks of mental imagery combined with eye movements can reduce the emotionality and vividness of both negative flashforwards and autobiographical memories, relative to a control condition of mental imagery alone [see Ref. (20)]. The main goal of the present study was to garner additional support for the effectiveness of this procedure by addressing two shortcomings identified in the experimental paradigms of earlier research. First, the majority of previous studies relied solely on self-report as a measure of emotionality. The present study aimed to develop a more objective measure of emotional intensity, which was achieved by testing whether a dual-task during mental imagery of a feared scenario would result in attenuated physiological responses. Second, in prior research the mental image rated during self-reports was formulated by the participant on each rating occasion, meaning that there was uncertainty over whether the same image was being assessed during pre- and post-test measures. This study drew upon a script-driven imagery procedure using audio recordings of a feared scenario to ensure that physiological responses and self-report ratings were focused on the same mental image on each measurement occasion.

The main finding of this study was that imagery with eye movements, which was the cognitively demanding task chosen, decreased HR response to the fear script from pre- to post-test measures. Self-report scores for emotionality demonstrated a small – but not significant – decrease for the experimental condition, whilst the control condition (imagery only) demonstrated a relative increase. These findings partially support the hypothesis that participants who have made eye movements whilst simultaneously holding a negative mental image in-mind will exhibit attenuated physiological reactivity toward the imagery depicted in the script. If HR is taken as a stand-alone assessment of emotionality [as in Ref. (50, 51)], then it can be said that these findings are consistent with prior research which found that dual-tasks cause reductions in emotional intensity for negative mental images. The current study also adds support to earlier findings (28) demonstrating that dual-tasks not only affect past memories, but also future-oriented mental images about feared scenarios.

The lack of significant results for SC and EMG measures may be partly attributed to the fact that measurements were taken over relatively long time periods (30 s), with such dimensions generally better suited to measuring immediate response to stimuli (52). It is possible that by averaging measures over this time period, any discrete differences in emotional valence may have been diminished. In regard to electrodermal response, this extended time period meant that SC level, which measures tonic change in electrical conductivity of skin, was used rather than the more informative measure of SC response measuring phasic change. As SC level generates a constantly moving baseline that is all the time changing within an individual, some researchers have suggested that as a measure it is difficult to derive and not overly informative (53, 54). Such problems have led to some researchers overlooking the standardized format for measuring psychophysiological responses used in Pitman and colleagues series of script-driven imagery studies [e.g., in Ref. (33–36)], and instead relying on HR alone as a measure of physiological reactivity to scripts [e.g., in Ref. (50, 51)]. In studies where significant differences in EMG and SC levels were found, the study format was generally comprised of a single measurement period comparing a clinical population, who demonstrated inflated levels of physiological reactivity, to non-clinical controls [e.g., in Ref. (12, 33, 35)].

In regard to gender, although our sample included a greater representation of females than males, this is reflective of the prevalence of public speaking anxiety in the general public. A number of studies to date have highlighted a fear of public speaking and social phobias more generally as having a higher prevalence amongst females (55–57). Thus, the fact that a larger proportion of females reached the inclusion threshold for the current study is unsurprising.

An additional finding in need of consideration was the fact that self-reports of emotional intensity were not affected by imagery with eye movements, in contrast to earlier research [e.g., in Ref. (28)]. The present study differed from previous research however in that, due to methodological reasons, mental imagery was based on a standardized fear script rather than encouraging participants to visualize a personalized fear scenario. As flashforwards related to a fear of public speaking are generally situation specific, with certain key details pertinent to an individual’s fear, this may have meant that what a participant finds to be most unpleasant about giving a presentation was missing from the script, resulting in low personal relevance and lower ratings. Supporting this theory are the self-report mean scores for emotionality of fear imagery, which in this study were lower than those in previous studies at the pre-test measure. For example, in Engelhard et al.’s (30) study examining flashforwards to feared future events in a non-clinical population, the mean rating participants gave for the emotionality of their personal fear imagery was 75; in the present study, the mean score was 65. This left less room for a decline in the subjective unpleasantness of mental images and could partially account for the measure failing to reach significance. For this reason, it is recommended that, in future research, individualized scripts targeting specific elements of fears should be formulated for each participant prior to partaking in the experiment. The possibility of irrelevant cognitive process and thoughts taking place during the imagery phase has also been noted, and may be addressed in future studies by including a manipulation check to verify that participants did indeed focus on the script presented to them during the imagery phase.

The use of a standardized fear script rather than personalized mental imagery also lends to the possibility that the underlying processes at play during the current study differed from those in previous studies. In the majority of prior research, participants were required to recall a mental image from long-term memory; either autobiographical or of a personal fear. Eye movements and other cognitively demanding tasks have been shown to result in the “blurring” of mental imagery through working memory taxation, and thus may alter these memories before their return to long-term store [e.g., in Ref. (20, 24)]. In the present study however, mental imagery was based upon a standardized fear script and so was recalled immediately after encoding. Memories can take several hours to solidify after initially being encoded, in what is referred to as a “consolidation period” [e.g., in Ref. (58)]. During this time, memories are subject to change, meaning it is possible that in this study eye movements interfered with consolidation rather than reconsolidation. van den Hout et al. (31) faced a similar issue in their study which demonstrated attenuation in the vividness of neutral pictures which were presented to participants for the first time during the experimental session. They deemed, however, that as memory performance was not affected and that the effects of the dual-task trial were still demonstrated, the underlying processes, though interesting, were not important to interpretation of the study. With the current study also demonstrating effects of dual-tasks on some dimensions of emotionality, it thus remains unclear whether an individualized script would have resulted in a different outcome. However, the possibility that distinct underlying processes exist for the effects of dual-tasks on long-term memories and recently encoded memories is something that may warrant future investigation.

The processes underlying the effects of dual-tasks are also of relevance when taking into consideration the self-report findings for vividness. Subjective vividness ratings were not expected to decrease in the present study due to the fact that participants listen to the fear script in its entirety for a second time after completing the mental imagery experiment, and before giving their post-test rating. As was outlined above, when a memory is recalled it is subject to interference, and listening to the fear scenario described in vivid terms during the post-test may have negated the blurring effects of the dual-task. If it can be shown that the post-intervention script-driven imagery procedure does indeed interfere with the effects of dual-tasks, an imagery period without a prior reading of the script could be one solution. Although this would again resurface the possibility that the mental image under consideration may differ between pre- and post-test measures, this option could be seen as the lesser of two evils if the effects of the dual-task are maintained. Script-driven imagery with corresponding psychophysiological measurements has the potential to be an invaluable assessment tool as an objective measure of the effectiveness of dual-task trials in future studies; however in order to establish a best practice method for use in the laboratory, it is important that this issue be resolved.^2^

An assumption of the working memory taxation theory is that demanding dual-tasks leave fewer resources available for maintaining a vivid mental image and for emotional reactions to it. These tasks typically lead to decreases in vividness as well as emotionality of the mental image, but some studies found only effects for vividness, and not emotional intensity (59), or the other way around (60). In the present study, attenuated HR response was demonstrated with no corresponding reduction in vividness. These findings raise interesting questions about how dual-tasks influence mental images. It has been hypothesized that decreased emotionality is a consequence of the decreased vividness of a mental image (25). However, another possibility is that it results directly from cognitive load. Research has shown that performing a cognitive demanding task modulates emotional responses in the brain: when task-load is increased, increased activity in the frontal cortex is associated with decreased activation in the emotional regions [amygdala and right insula; (61)]. For both theoretical and clinical reasons, an important direction for future research is to investigate the underlying mechanisms linking dual-tasks to effects on emotional mental images. This knowledge can be used to further optimize the intervention. A limitation of the current study was that we did not measure the effects of the dual-task intervention on public speaking fear with a clinical scale. Another important direction for future research is to test whether the intervention has direct effects on fear/anxiety. Nevertheless, as noted by Hackman and Holmes (62), it may be very useful to have a therapeutic tool that could at least initially reduce emotionality, as this may facilitate a patient’s willingness to engage in exposure therapy. A dual-task could be such a “cognitive performance aid” (63) that targets past or future-oriented aversive images related to public speaking anxiety and other anxiety disorders.

## Conclusion

In summary, although further work is required to perfect a script-driven imagery procedure suitable for use in combination with dual-task interventions, the current study produced some interesting findings. The fact that HR response to fear scripts resulted in a significant decrease from pre- to post-test measures proves that the effects of dual-tasks extend beyond the parameters of self-report. Furthermore, the present study provides additional support to previous findings demonstrating the utility of dual-tasks for future-oriented fear imagery as well as past negative memories. Given that future-oriented anxieties such as public speaking fear represent a substantial portion of clinical populations, these findings are encouraging. Popular interventions for combating public speaking fear are currently heavily based on either exposure, which can be distressing for the individual, or on skills training, which demonstrates little success in targeting avoidance (4). If long-term clinical effects of dual-tasks can be demonstrated in future research, this relatively non-evasive intervention could be seen as a viable alternative to established treatment methods, with positive implications for those struggling with this restrictive fear.

## Funding

This study was funded by the Netherlands Organization for Scientific Research (NWO) with Vidi grant 452-08-015 to IE.

## Conflict of Interest Statement

The authors declare that the research was conducted in the absence of any commercial or financial relationships that could be construed as a potential conflict of interest.

## Footnotes

^1^McCroskey (40) advise that a score of 24 or higher generally denotes a high fear of public speaking, and a score of lower than 14 generally denotes a low fear of public speaking. Because we were interested in anxious apprehension of public speaking (and not just high levels), we decided to use the cut-off score of 20. Eight participants with PRCA-24 Public Speaking sub-scores ranging from 20 to 23 were found to rate the public speaking script as being sufficiently unpleasant to warrant a lowering of the inclusion criterion for the main experiment, with a mean rating of 80 on a 100 mm visual analog scale.

^2^This is primarily a problem for experimental laboratory studies, as patients receiving EMDR treatment in accordance with the guidelines set out by Shapiro (64) undergo eye movements for a period that far exceeds any lab-based studies, and on multiple occasions. Therefore, the treatment effects are a lot more durable and long-lasting, and are unlikely to be affected by listening to a script reading.
